# Dr. W. G. A. Bonwill

**Published:** 1899-11

**Authors:** 


### Obituary.

### DR. W. G. A. BONWILL.

A BRIEF announcement of the death of Dr. Bonwill was given in our last issue as it was being prepared for the press, and too late for an extended notice.

His death occurred on September 24, 1899, through uræmia, the result of acute prostitis, after an illness of seven weeks.

Dr. Bonwill's early life was spent in Delaware. He commenced the practice of dentistry in Dover, in that State, in 1854, and remained there some years, subsequently removing to Philadelphia, where he enjoyed a large practice up to the period of his last sickness.

It was during this period that he carried on the work that gave him an international reputation, and it is that period and the work evolved that has invested his life with an interest to all practitioners of dentistry.

The universal thought is predominant in the human mind that death ends all antagonisms, and leaves only the most kindly feelings to influence the last word at the open grave. This is not only proper, but it evidences the best in man that he is willing to permit the record of a life, whether good or ill, to pass beyond the range of human criticism.

When a good man dies it is due him that his life work be calmly but truly given, that the lesson may be of value to others, for it is the good in man that lives and becomes an educational force. Every life that contributes something to the uplifting of humanity is part of the foundation upon which the true hope for mankind is concentrated and which makes for the progress of the world.

Dr. W. G. A. Bonwill was one of the remarkable men of his day and generation. He was a man among men, isolated largely from his fellows by the peculiarities of inheritance, but ever maintaining an unselfish devotion to the profession of his choice, ever ready to give his original ideas to an unbelieving world, and ever ready to defend those ideas even though it separated him from those he respected. He truly was one who had "trodden the wine press alone," and illustrated more fully in his own experience, as but few others, the old biblical quotation, "A prophet is not without honor, save in his own country." Abroad he was welcomed and sought after as the one American worthy of honor, while at home he worked and lived his life almost alone in a professional sense.

It is unnecessary here to enter into an explanation of this singular antagonism. Suffice it to say it existed, and was mainly due to his own peculiarities and a lack of appreciation of the sterling worth of the man by those with whom he was surrounded. The very few who had the privilege of entering into his inner nature were able to throw aside the mental characteristics that measurably marred his life, and had revealed to them an underlying spirit of helpful devotion to the best interests of dentistry and an unselfish desire to aid humanity. He was ever ready, in season and out of season, to assist a struggling practitioner. In this respect he excelled all men in his profession in his own line of work. The superficial observer charged this to an overmastering desire for personal glory, but those who knew him intimately were aware of the fact that he was so thoroughly convinced that his methods were true and helpful that he was willing to sacrifice himself to bring others to the same standard. No other man travelled farther or spared himself less in demonstrating his ideas than Dr. Bonwill, and it has not been given to many men to prove to an unbelieving generation that, in the main, he was correct.

He was a born mechanic. By this is not meant that his mind was capable of perfecting in every detail his original ideas. It appeared to the writer that he lacked in this respect, and as a result, many of his appliances were crudely evolved from his active brain at their inception, and thus offered an opportunity for others to attempt improvements and to deprive him of the honor justly belonging to his work.

When Dr. Bonwill first located in Philadelphia he was confirmed in the belief that the method of using cohesive foil was a mistake, and the writer was severely criticised by him for teaching this to the students in his charge. His thorough belief in non-cohesive, or, as it was then called, "soft foil," was so pronounced that he did not hesitate to denounce the practice of those who use the more modern form. When he became a convert to the use of cohesive gold, which naturally followed his invention of the electric mallet, he became equally the antagonist of all who continued the use of non-cohesive gold, and carried this feeling up to the last days of his life. He seemed incapable of recognizing that there is a middle ground for the liberal mind to rest. This tendency to extreme views embittered many against him and disturbed friendly relations with those whose friendship would have been helpful and mentally invigorating.

The invention of the electric mallet first brought Dr. Bonwill into prominence. Other attempts to apply power in the use of the mallet had preceded his effort, but not in this direction. The electric, when he finally succeeded in perfecting it, entirely superseded these, and for a time held undisputed sway with the best operators of the period. The evolution of this instrument illustrates the previous remark that nothing seemed to issue from his brain fully developed. The various stages of its growth, which were familiar to the writer and which have been preserved by the inventor, demonstrate this very conclusively. After, however, some two years of effort the crude original conception was reduced to a reasonably good working instrument. It revolutionized the ideas, previously prevailing, of the hammer-blow. It was discovered that the rapidity of the blow delivered by this instrument overcame mobility, and thus secured a compaction of the gold not obtainable by the heavy mallets previously used. This quality of the electric, in the opinion of the writer, has never been reached by any of the mechanical mallets, not even the Bonwill, the best of all that class of instruments.

The history of the introduction of this mallet has never been written, and perhaps it is just as well to let it sink into forgetfulness, but its introduction engendered a crop of bad feelings that were never exterminated or softened during Dr. Bonwill's life. This was partially due to his unwillingness to recognize the fact that any other mind could, in the least degree, improve upon his work, and, on the other hand, a very great wrong was done him by stamping his invention with another man's name. This was a most discreditable proceeding, and for a time it almost obliterated that of the inventor from the instrument. There were other wrongs attempted, but these were not very successful in depriving him of the honor of this invention. This failure was mainly due to the fact that the dental profession indignantly took sides with the inventor, and made it impossible for any one to supplant him in the honor due him, but it took years to accomplish this. At the present time no one has the temerity to dispute the fact that Bonwill originated the instrument and made it a practical working force.

The antagonisms met with in its introduction led his active mind to evolve a mechanical power that would entirely obviate the necessity for the use of a battery. This he, in part, succeeded in accomplishing in the invention of what is known as Bonwill's mechanical mallet. While a number of others have endeavored to originate instruments based on a mechanical blow, Bonwill's invention remains the simplest in construction and the most practical. In his own opinion it superseded the electric, but this view is not held by those equally well qualified to judge of their respective merits; in fact, the blow the mechanical delivers cannot compare with that of the electric, at least not when applied with foot-power.

The evolution of the so-called dental engine changed the whole character of operative dentistry, and in this development Dr. Bonwill took a conspicuous part, and after many efforts he succeeded in giving dentistry a very practical and simple cord engine, which, with the hand-piece, reduced the labor of preparing cavities and finishing fillings. While this instrument has not entirely superseded other forms, it still is preferred by the majority. The studies of Dr. Bonwill in this direction led him to adapt this instrument to certain surgical operations, and the "surgical engine" was the result. This, as it has since been perfected, is, in the opinion of some, only second in importance to the discovery of anæsthesia. It requires, of course, skill in handling tools, but its value has been so frequently demonstrated that criticism has lost all its force. It is this, perhaps, more than anything else, that gave Dr. Bonwill the respect and honor of the first surgeons in this country and abroad. When its value is properly appreciated, and skill in its use attained, the old, crude, and essentially barbarous methods will have been cast aside and relegated to the museums as relics of a by-gone and ignorant age.

His method of "rapid breathing" as an analgesic never was adopted except by the few, although it was fully endorsed by very high authority, and has been proved in many cases by himself and others as having a positive value in minor surgical operations.

Dr. Bonwill was severely criticised for claiming almost everything introduced. While he deserved this, it must be said in extenuation that he very frequently made good this claim by actual proof. His active mind was ever on the alert for new methods, and he naturally attempted and abandoned many things that were eventually perfected by others.

Space will not permit the writer to follow this original thinker through all the ideas that he moulded into form, but the history of his work would be incomplete if no allusion were made to that on articulation. He began this by constructing an articulator based, as he believed, on the natural movements of the human jaw. Strange to say, for more than twenty years this admirable instrument was allowed to sink into oblivion and neglected by almost all workers upon artificial plates. Slowly, however, its value became recognized by the few, but even at the present time it has not entered into general use. Why this should be it is difficult to determine, for it is beyond question the only true scientific articulator ever invented.

Upon this instrument he based his theories antagonistic to evolution as understood by Darwin and his followers. With all the energy of his nature, he forced attention to his ideas, and at no time was he discouraged by the antagonisms met with. His efforts to prove his position were not very successful, but he was always ready to enter the arena of disputation, in defence of his propositions, with the best scientific minds in and out of his profession. While he regarded this as the most important work of his life, it is presumed it will die with him, for his theories found few who would seriously entertain them. His general principles of articulation will remain as a portion of that living monument which he builded in the minds of his professional brethren.

He had in his teeming brain many things which, he informed the writer, he proposed to develop, and had he lived a few years longer these would, doubtless, have been evolved and perfected.

His last clinic was given at the meeting of the Pennsylvania State Dental Society, held at Neversink Mountain in July last. It was one of his best, and he fully enjoyed it, stating afterwards that "the boys treated him kindly."

There was much in this last remark indicative of his character. No man living was more sensitive to opposition, or more ready to meet it with equal bitterness, and no one was more amenable to every expression of kindness. The writer has often seen the tears well up in his eyes at the mere mention of some kind remark made by a professional brother. It was this inner life that so few knew, but it was this that drew that very few closer to him in spite of his peculiarities.

As the writer looked at his placid face as he lay in his coffin, he could not help the feeling that at last this ever-active body was at rest, but if we believe in the immortality of the animating spirit, it is impossible to think of its resting anywhere in the great universe of mind.

W. G. A. Bonwill has closed his work on earth, the harvest for him has been gathered, but he has left broken sheaves for others to bring together for the nutrition of our professional life. He lived that his profession might be benefited by his work, and he truly died a martyr to his faith, for the continued strain under which he lived, mental and physical, undoubtedly shortened a life which had every promise of continued development.

At the open grave we can bury our prejudices and our antagonisms, and truly say a great man has been gathered all too early to the last resting place on earth, and as we lay up the treasures of his life, may they be continual incentives to follow him unselfishly to the end. He has built his own monument, and in the cycles of the future his name will be remembered when others who have labored with equal assiduity have passed into oblivion.

