# Correction

**Published:** 1899-12

**Authors:** 


### CORRECTION.

In the obituary article on Dr. Bonwill, in the November number, two letters dropped out of the word *prostatitis*, to the annoyance of the editor. The intelligent reader no doubt supplied the omission.

